# Closed Genome Sequence of *Octadecabacter temperatus* SB1, the First Mesophilic Species of the Genus *Octadecabacter*

**DOI:** 10.1128/genomeA.01051-15

**Published:** 2015-09-10

**Authors:** Sonja Voget, Sara Billerbeck, Meinhard Simon, Rolf Daniel

**Affiliations:** a Genomic and Applied Microbiology and Göttingen Genomics Laboratory, Georg-August-University, Göttingen, Germany; b Institute for Chemistry and Biology of the Marine Environment, University of Oldenburg, Oldenburg, Germany

## Abstract

The Gram-negative alphaproteobacterium *Octadecabacter temperatus* SB1 (DSM 26878) belongs to the marine *Roseobacter* clade. The genome of this strain is the smallest closed genome of the *Roseobacter* clade. *O. temperatus* SB1 is the first described nonpolar mesophilic isolate of the genus *Octadecabacter* and the type strain of the species.

## GENOME ANNOUNCEMENT

The *Roseobacter* clade (family *Rhodobacteraceae*) is a group of exclusively marine bacteria within the alphaproteobacteria. In sea ice microbial communities (SIMCO), roseobacters are represented by the genus *Octadecabacter*. *O. antarcticus* constitutes up to 1% of the total bacterial community in southern ocean SIMCOs, while *O. arcticus* comprises up to 23% of the total bacterial community in Arctic SIMCOs (1). The nonpolar clusters are represented only by 16S rRNA gene sequences from uncultured organisms (2). A recently described mesophilic species has been reclassified as *Pseudooctadecabacter jejudonensis* (3, 4). *Octadecabacter temperatus* SB1 (DSM 26878) was isolated from a surface water sample collected in the harbor of the island of Helgoland in the North Sea (4). It is the first described nonpolar mesophilic isolate of the genus *Octadecabacter* and the type strain of the species. The strain was grown in marine broth (MB) medium at 20°C (4). Chromosomal DNA was isolated with the MasterPure complete DNA purification kit as recommended by the manufacturer (Epicentre, Madison, WI, USA). Whole-genome sequencing was performed with a combined approach using the 454 GS-FLX + pyrosequencer (Roche Life Science, Mannheim, Germany) and the Genome Analyzer IIx (Illumina, San Diego, CA, USA). Shotgun libraries for 454 sequencing as well as Nextera XT paired-end libraries for Illumina sequencing were prepared according to the manufacturer’s protocols. Sequencing resulted in 54,153 pyrosequencing reads and 5,626,412 paired-end Illumina reads. Hybrid assembly of Illumina and 454 data was performed *de novo* with Mira v3.4.0.1 (5). Illumina data was also assembled with SPAdes v2.5.0 (6). Both assemblies were combined resulting in 8 contigs and a 158-fold coverage. Remaining gaps were closed by PCR-based techniques and Sanger sequencing of the recovered products using BigDye 3.0 chemistry on an ABI3730XL capillary sequencer (Applied Biosystems, Life Technologies GmbH, Darmstadt, Germany), and by employing Gap4 v4.11 of the Staden Package (7). The closed genome sequence of *O. temperatus* SB1 consists of one chromosome (3.23 Mb) and one plasmid (31.6 kb). To date this genome represents the smallest closed genome within the *Roseobacter* clade. Protein-encoding genes were identified and annotated with the Prokka annotation pipeline using Prodigal v2.6 (8). The closed genome harbored 1 rRNA operon, 39 tRNA genes, and 3,284 potential protein-encoding genes, of which 2,711 have a predicted function. The plasmid encodes a replication system of the RepABC-type with highest similarity to the RepC9-subtype, which is also present in the plasmids of the polar *Octadecabacter* species (9). Moreover, genes for the biosynthesis and export of lipopolysaccharides are encoded by the plasmid. Like the polar *Octadecabacter* species, *O. temperatus* SB1 lacks a gene cluster for aerobic anoxygenic photosynthesis. In addition, genes encoding xanthorhodopsins are absent in the genome of *O. temperatus*. This is in contrast to the polar *Octadecabacter* species, which harbor such genes as putative adaption for living in icy environments (2).

### Nucleotide sequence accession numbers.

This whole-genome shotgun project has been deposited at DDBJ/EMBL/GenBank under the accession numbers CP012160 (chromosome) and CP012161 (plasmid).
